# Effects of Oral Supplementation with Myo-Inositol and D-Chiro-Inositol on Ovarian Functions in Female Long-Term Survivors of Lymphoma: Results from a Prospective Case–Control Analysis

**DOI:** 10.3390/jpm12091536

**Published:** 2022-09-19

**Authors:** Miriam Dellino, Eliano Cascardi, Claudia Leoni, Francesca Fortunato, Annarita Fusco, Raffaele Tinelli, Gerardo Cazzato, Salvatore Scacco, Antonio Gnoni, Antonio Scilimati, Vera Loizzi, Antonio Malvasi, Anna Sapino, Vincenzo Pinto, Ettore Cicinelli, Giovanni Di Vagno, Gennaro Cormio, Vito Chiantera, Antonio Simone Laganà

**Affiliations:** 1 Department of Biomedical Sciences and Human Oncology, University of Bari, 70121 Bari, Italy; 2 Clinic of Obstetrics and Gynecology, “San Paolo” Hospital, 70132 Bari, Italy; 3 Department of Medical Sciences, University of Turin, 10124 Turin, Italy; 4 Pathology Unit, FPO-IRCCS Candiolo Cancer Institute, 10060 Candiolo, Italy; 5 Institute of Biomembranes, Bioenergetics and Molecular Biotechnologies, Consiglio Nazionale delle Ricerche, 70126 Bari, Italy; 6 Department of Medical and Surgical Sciences, University of Foggia, 71122 Foggia, Italy; 7 Department of Obstetrics and Gynecology, University Medical School of Bari, 70121 Bari, Italy; 8 Department of Obstetrics and Gynecology, “Valle d’Itria” Hospital, 74015 Martina Franca, Italy; 9 Department of Emergency and Organ Transplantation, University of Bari “Aldo Moro”, 70121 Bari, Italy; 10 Department of Basic Medical Sciences and Neurosciences, University of Bari “Aldo Moro”, 70121 Bari, Italy; 11 Department of Pharmacy—Pharmaceutical Sciences, University of Bari “Aldo Moro”, 70121 Bari, Italy; 12 Interdisciplinar Department of Medicine, Obstetrics and Gynecology Unit, University of Bari “Aldo Moro”, 70121 Bari, Italy; 13 Gynecologic Oncology Unit, IRCCS Istituto Tumori Giovanni Paolo II, Department of interdisciplinary Medicine (DIM), University of Bari “Aldo Moro”, 70121 Bari, Italy; 14 Unit of Gynecologic Oncology, ARNAS “Civico—Di Cristina—Benfratelli”, Department of Health Promotion, Mother and Child Care, Internal Medicine and Medical Specialties (PROMISE), University of Palermo, 90127 Palermo, Italy

**Keywords:** precision medicine, health promotion, Inositol, lymphoma, fertility preservation, reproductive outcomes

## Abstract

The progressive improvement of lymphoma treatment has led to an important prolongation of patient survival and life expectancy. The principal international scientific societies of oncology now therefore recommend that long-term survivors of lymphoma join fertility programs. Specifically, fertile-age patients should be assisted by a multidisciplinary team, including specialists dedicated to fertility preservation in oncology, in order to support the completion of their reproductive project. In the general population, the use of Myo-Inositol and D-Chiro-Inositol (MI/DCI) has been demonstrated to be an effective choice to treat ovarian dysfunctions, with a consequent improvement in reproductive outcomes, so it may represent an adjuvant strategy for this purpose. We therefore conducted a pilot prospective case–control study to evaluate the potentialities of this nutritional supplement, with the aim of optimizing reproductive function in female long-term survivors of lymphoma. One group underwent oral supplementation with MI 1200 mg and DCI 135 mg per day for 12 months, compared with controls who underwent no treatment in the same period. After 12 months, FSH, LH, and progesterone levels, as well as oligomenorrhea and antral follicle count (AFC), were significantly improved in the MI/DCI group. In addition, a significantly higher mean value in FSH and LH and a significantly lower mean AFC value in the right ovary were observed in controls compared to the MI/DCI group. Despite the need for further investigation, MI/DCI could be considered a potential adjuvant strategy to restore ovarian function in female long-term survivors of lymphoma.

## 1. Introduction

Anticancer regimens used to treat women with lymphoproliferative neoplasms are known to cause transitory or permanent damage to their reproductive function [1]. The rates of gonadotoxicity of each chemotherapy could be modulated according to the dose intensity, number of cycles, and type of agent or combination [2]. Classical Hodgkin lymphoma (cHL) and Diffuse Large B-Cell Lymphoma (DLBCL), including Primary Mediastinal Large B-Cell Lymphoma (PMBL), usually have a higher incidence in the second or third decade of life, and present cure rates of more than 75% [3,4]. These histotypes need a poli-chemotherapy with adriamycin, bleomycin, vinblastine, and dacarbazine (ABVD), which involves the association of alkylating and non-alkylating agents [5]. 

An autologous hematopoietic stem cell transplant (ASCT) is needed in approximately 10% of cHL and 40% of DLBCL cases, and has a worse impact on female fertility compared to poli-chemotherapy [6]. Therefore, both chemotherapy (reducing the primordial follicle pool and then the ovarian reserve) and radiotherapy of the pelvic area could put the patient at risk of gonadotoxicity [7]. In this scenario, data concerning fertility in cHL have been mainly investigated by the German Hodgkin Study Group (GHSG) and the European Organization for Research and Treatment of Cancer (EORTC) through amenorrhea and anti-Müllerian hormone (AMH) evaluation [8]. Indeed, amenorrhea has been used in numerous studies to assess the gonadotoxicity of oncological treatment and reflect, at least in part, ovarian function [9]. Accumulating evidence suggests that after early-stage disease treatment (2–4 cycles), more than 90% of patients report a low gonadotoxic effect, with a regular menstrual cycle after therapy (median recovery of 1 year) [10]. For advanced-stage diseases (treated with six courses of ABVD), the gonadotoxic effect varies according to age [8]. Indeed, a complete AMH restoration during the 3-year follow-up period was detected in all women under 35 years, but only in 37% of older ones [9]. Moreover, in a Cochrane meta-analysis, gonadotropin-releasing hormone agonist (GnRHa) seemed to be effective in shielding the ovaries during chemotherapy, in terms of treatment-related premature ovarian failure, ovulation, and menstruation recovery [11]. In addition to ovarian suppression with GnRHa, there are several well-established procedures (oocyte cryopreservation and ovarian cortex cryopreservation for women, and sperm cryopreservation for men) that could prevent the detrimental effects of these therapies in young patients [11]. Nowadays, considering the increased life expectancy of long-term survivors of lymphoma (patients in complete remission of the disease for more than 5 years), a fertility program with multidisciplinary counselling is indicated [12,13]. In particular, in order to assess the possible damage to the reproductive organs after anticancer therapies, international scientific societies such as the American Society for Reproductive Medicine (ASRM), the American Society of Clinical Oncology (ASCO) [14], the European Society for Medical Oncology (ESMO), and the International Society for Fertility Preservation (ISFP) [14] strongly recommend personalized and multidisciplinary counseling for each patient [15,16]. In particular, the surveillance programs for the management of long-term lymphoma survivors should involve a team with onco-hematologists, radiotherapists, cardiologists, an endocrinologist, a psychologist, a gynecologist, a urologist, and a nutritional biologist [14]. The goal is to provide individualized therapeutic possibilities to restore endocrine function [17]. Following these recommendations, an outpatient service dedicated to fertile patients with oncological disease was established in our Clinic of Gynecology Oncology, aiming to perform a careful assessment of patients’ fertility state and support their reproductive project. Considering the potential adverse effects of anticancer treatments on ovarian function, oral supplementation with Myo-Inositol and D-Chiro-Inositol (MI/DCI) may be considered an adjuvant strategy [15]. Indeed, over the years, MI/DCI has established its validity alongside the classic pharmacological therapies, with robust compliance, safety, and effectiveness. In particular, MI/DCI has shown great potential in counteracting ovarian dysfunction and optimizing reproductive function [15].

Considering this scenario, we designed and performed a prospective, single-center, case–control study aiming to evaluate the effects of oral supplementation with MI/DCI on ovarian function parameters, menstrual cycle characteristics, dysmenorrhea, and dyspareunia in long-term survivors of lymphoma.

## 2. Materials and Methods

**Figure 1:**
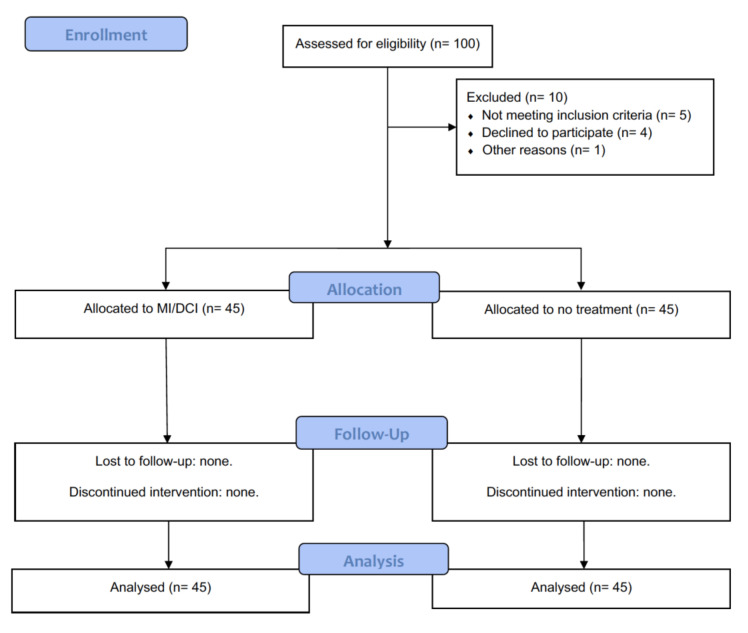
Flow-chart for inclusion and treatment of long-term survivors of lymphoma. MI/DCI: Myo-Inositol and D-Chiro-Inositol oral supplementation.

From January 2020 to January 2021, 90 female patients, all long-term survivors of lymphoma with an average age of 34 years (range 25–44), were considered eligible and enrolled in the study (Figure 1). Each patient was informed about the procedures and gave their informed consent to allow data collection for research purposes. The design, analysis, interpretation of data, drafting, and revisions conformed to the Helsinki Declaration, the Committee on Publication Ethics (COPE) guidelines, and the Reporting of studies Conducted using Observational Routinely Collected Data (RECORD) statement [18], and are available through the Enhancing the Quality and Transparency of Health Research (EQUATOR) network. The study was registered on ClinicalTrials.gov (ID: NCT05410314). Considering that data analyzed in this study were collected during routine clinical activity and fully anonymized, and that investigators did not perform any interventional procedure, formal Institutional Review Board approval was not required due to the observational nature of the study. It was not advertised, and no remuneration was offered to the patients to enter or continue the study. An independent data safety and monitoring committee evaluated the ad interim and final results of this study. Over the study period, there were no significant differences in the facilities available for patient care and in the referral patterns of our service.

We included patients who met the following criteria: (1) diagnosis of cHL or DLBCL; (2) previous treatment with ABVD + radiotherapy (without under diaphragmatic and/or pelvic irradiation); (3) no relapse during quinquennial follow-up. We excluded women who underwent previous under diaphragmatic and/or pelvic irradiation, as well as women with any other endocrinological, autoimmune, and/or metabolic disease that could affect the investigated outcomes.

Patients enrolled in this study were cHL (85%) and DLBCL (15%) long-term survivors, treated mainly with ABVD + radiotherapy (without under diaphragmatic and/or pelvic irradiation), without relapse during quinquennial follow-up. We conducted a prospective case–control study, subdividing the population into two groups: the first (A group) underwent oral supplementation with MI/DCI for 12 months; the second group (B group) underwent follow-up without any nutritional supplement for 12 months. The choice to undergo oral supplementation with MI/DCI or no treatment, and hence the assignment to one of the two groups, was based on the patient’s choice and preference, without any influence by the investigators. The MI/DCI product used in our clinical study was Ovofert Fast ® oral capsules (CR.L PHARMA Esadea S.R.L.—Taranto), containing MI 400 mg and DCI 45 mg in each capsule. The dose per day in the treatment group was 3 capsules per day (early morning, mid-day, and late evening), for a total of 1200 mg of MI and 135 mg of DCI per day. Patients in the treatment group were advised to take oral capsules 2 h before or after main meals, in order to achieve the best absorption of the molecules, based on previous pharmacokinetic analyses [19]. Both groups were followed up for 12 months, at T0 (during first evaluation) and T12 (after twelve months), with a blood test for AMH (ng/mL). Moreover, between the third and fifth day of the menstrual cycle, we dosed also FSH (mIU/mL), LH (mIU/mL), and 17-β estradiol (pg/mL); progesterone (PG; ng/mL) was dosed on day 21 of the menstrual cycle. Additionally, a transvaginal ultrasound scan at both T0 and T12 was performed by the same sonographer (M.D.), using a 5–9 MHz endo-cavitary volumetric probe and a Samsung WS80A ultrasound system. Patients were positioned for the exam in the dorsal lithotomy position and with an empty urinary bladder. The probe was gradually introduced into the vagina to obtain a suitable image for measurement of the antral follicle count (AFC) for each ovary. In addition, we recorded clinical data about menstrual frequency (oligomenorrhea defined as menstrual cycle <25 days, normal menstrual cycle between 25 and 36 days, polymenorrhea if >36 days), the duration of the cycle (defined as short if <3 days, normal 3–7 days, long >7 days, respectively), the amount of the menstrual cycle (minimal if <30 mL, normal 30–80 mL, >80 mL abundant), and dysmenorrhea and dyspareunia (defined according to a visual analogue scale—VAS—to measure pain, from 0, which refers to no pain, to 10, which refers to the maximum pain; mild pain was defined by VAS ≤ 7; severe pain by VAS > 7).

### Statistical Analysis

Categorical variables were expressed as counts and percentages in each category, and continuous variables as means ± standard deviation. Direct inter-group and intra-group comparisons of the menstrual frequency, menstrual duration, menstrual quantity, dyspareunia, and dysmenorrhea were carried out in a univariate analysis using the chi-square test (χ2) and the odds ratio (ORs) with 95% Cis or Fisher’s exact test. Differences in continuous variables were tested with Student’s *t* test for normally distributed ones, or the Mann–Whitney U test when variables showed a non-normal distribution. The level of statistical significance was set at *p* ≤ 0.05. Analysis was conducted with STATA/SE 15.0 (StataCorp, 2017), R software (version 4.0.1).

## 3. Results

Patients enrolled in this study received the first diagnosis of lymphoma at a range of 14–41 years old (an average age of 27 years; cHL 85% and DLBCL 15%) and were long-term survivors.

In group A (long-term survivors of lymphoma who underwent MI/DCI therapy, Table 1), a significant reduction after 12 months was observed for FSH (*p* = 0.0199), LH (*p* = 0.0219), and oligomenorrhea (*p* < 0.0001) and a reduction in the limits of statistical significance for PG level (*p* = 0.0501); in addition, the AFC of the right ovary at T12 increased significantly (*p* = 0.0055). In group B (long-term survivors of lymphoma without MI/DCI as nutritional supplement, Table 2), a significant reduction after 12 months was observed for oligomenorrhea (*p* = 0.0023), and a significant worsening was observed for dyspareunia (*p* = 0.0001) and dysmenorrhea (*p* < 0.0001). None of the other ovarian function parameters (Table 1), menstrual cycle characteristics, dysmenorrhea, or dyspareunia (Table 2) showed significant differences between baseline (T0) and after 12 months (T12).

The inter-group comparison at baseline (Table 3) showed a higher mean value of the AFC of the right ovary in group B compared to group A (*p* = 0.0483); conversely, the mean value of AFC of the left ovary was lower in group B with respect to group A (*p* = 0.0406). In addition, in group A, the proportion of oligomenorrhea was higher compared to group B (*p* = 0.0114). After 12 months (Table 3), a significantly higher mean value in FSH (*p* = 0.0047) and LH (*p* = 0.0102), and a significantly lower mean AFC value in the right ovary, were observed in group B compared to group A (*p* = 0.0403). Moreover, a significant increase in dyspareunia (*p* = 0.0031) and dysmenorrhea (*p* < 0.0001) was observed in group B compared to group A (Table 4). None of the other ovarian function parameters (Table 3), menstrual cycle characteristics, dysmenorrhea, or dyspareunia (Table 4) showed significant differences in the inter-group comparison at baseline (T0) and after 12 months (T12).

## 4. Discussion

To the best of our knowledge, this represents the first pilot study aiming to evaluate ovarian function parameters in long-term survivors of lymphoma with or without MI/DCI treatment for 12 months. To date, several studies have reported that MI/DCI plays a pivotal role for the physiology of female and male reproduction, since Inositol is involved in several mechanisms, such as cellular energetic metabolism, cellular motility, insulin signaling, and steroidogenesis [20]. Specifically, in women, MI represents an FSH second messenger and orchestrates FSH-mediated pathways that control the proliferation and maturation of granulosa cells [21]. Based on this role, MI has a crucial role in oocyte maturation and in achieving good-quality oocytes and embryos [17]. Indeed, accumulating evidence suggests that the physiological ovarian status depends on a correct ratio of MI/DCI concentrations [22]. According to our data analysis, comparing ovarian function parameters between baseline (T0) and after 12 months of oral supplementation with MI/DCI, we found a significant reduction in FSH and an increase in PG and AFC of the right ovary. This result could be due, at least in part, to the known effect of MI/DCI on ovulation improvement, which contrasts with luteal insufficiency, typical in these patients [16]. Nevertheless, MI/DCI does not seem to have an impact on ovarian reserves, since AMH values were not significantly different at the end of the treatment. Moreover, in the control group, we did not find significant differences in hormonal levels between baseline and after 12 months of follow-up without any dietary supplement. We also take the opportunity to highlight, as corollary results, that the controls showed adequate ovarian reserves (i.e., AMH levels and AFC), confirming acceptable fertility potential in young female long-term survivors of lymphoma [23]. Among the most important results, we found that the menstrual cycle, as well as dyspareunia and dysmenorrhea, appear to be improved in patients under MI/DCI treatment, despite the absence of significant hormonal changes. This point is of paramount importance, considering that women treated with MI/DCI had a high rate of oligomenorrhea at baseline (T0). Consequently, this result confirms that MI/DCI supplementation can optimize gonadic performance and reduce ovarian dysfunction symptomatology [23].

Despite our results, several limitations should be considered for a proper data interpretation: first of all, the lack of previous similar studies does not allow a direct comparison with other clinical experience; second, we enrolled a limited number of women, so the study may be underpowered to detected minimal differences among the investigated parameters; third, we followed up with the patients for a relatively short period of time (12 months); finally, patients were not randomized to take or not take the treatment—we did not design the study with a placebo-controlled arm and so it was not possible to blind investigators and participants to the treatment allocation.

We therefore solicit further studies on lager cohorts to confirm our preliminary findings in a larger setting that may allow greater reliability, and to identify the timing of the effect of MI/DCI in terms of fertility and pregnancy rate. In addition, future investigations should aim to evaluate the effects of different combinations/doses of MI and DCI, ideally using a randomized, placebo-controlled, double-blind clinical trial, on ovarian function parameters and menstrual cycle characteristics.

Considering the pivotal role of MI/DCI in the signaling of insulin, the dosage of blood glycemia, insulin, Homeostatic Model Assessment (HOMA) index, body mass index, and nutritional evaluation could also be integrated into a protocol for the multivariate analysis of results [24]. Moreover, based on the growing evidence of the effectiveness of MI/DCI on sperm function [23,25,26,27,28,29,30,31], its effects in male long-term survivors of lymphoma could be tested. 

## 5. Conclusions

In conclusion, considering the safety of MI/DCI, new scenarios and recommendations for fertility care for young oncological survivors could be realized in the future.

## Figures and Tables

**Table 1:** Comparison of ovarian function parameters in long-term survivors of lymphoma between baseline (T0) and after 12 months of oral supplementation with Myo-Inositol and D-Chiro-Inositol combined therapy (T12; Group A) and between baseline (T0) and after 12 months of follow-up without any nutritional supplement (T12; Group B).

Intra-GroupComparison	Group A					Group B				
	T0		T12		*p*	T0		T12		*p*
	Mean (SD)	CI 95%	Mean (SD)	CI 95%		Mean (SD)	CI 95%	Mean (SD)	CI 95%	
AMH (ng/mL)	4.23 (2.38)	3.51–4.95	4.3 (2.13)	3.65–4.94	0.4465	4.45 (2.74)	3.62–5.28	4.64 (2.64)	3.85–5.44	0.3700
FSH (mIU/mL)	10.08 (7.64)	7.77–12.38	7.15 (5.43)	5.52–8.78	0.0199	9.94 (7.50)	7.68–12.19	10.44 (6.31)	8.55–12.34	0.3654
LH (mIU/mL)	14.43 (8.22)	11.95–16.90	11.20 (6.64)	9.21–13.20	0.0219	14.8 (8.40)	12.27–17.32	15.76 (11.11)	12.42–19.10	0.3220
17-β Estradiol (pg/mL)	107.57 (45.86)	93.79–121.35	100.31 (54.89)	83.81–116.80	0.2487	109.75 (47.45)	95.49–124.01	111.83 (51.66)	96.31–127.35	0.4214
PG (ng/mL)	9.47 (4.32)	8.17–10.77	11.13 (5.09)	9.60–12.66	0.0501	11.18 (5.55)	9.51–12.85	10.92 (4.93)	9.44–12.40	0.4090
AFC of right ovary	6.22 (2.89)	5.35–7.09	7.8 (2.86)	6.93–8.66	0.0055	7.61 (4.72)	6.18–9.03	6.75 (2.73)	5.93–7.58	0.1487
AFC of left ovary	7.58 (2.64)	6.78–8.31	6.93 (2.61)	6.15–7.72	0.1240	6.57 (2.73)	5.75–7.39	7.02 (2.57)	6.25–7.79	0.2146

AMH: anti-Müllerian hormone; FSH: follicle-stimulating hormone; LH: luteinizing hormone; PG: progesterone; AFC: antral follicle count.

**Table 2:** Comparison of menstrual frequency, duration of the cycle, the amount of the menstrual cycle, dysmenorrhea, and dyspareunia in long-term survivors of lymphoma between baseline (T0) and after 12 months (T12) of follow-up with (Group A) and without nutritional supplement (Group B).

	Group A		Univariate Analysis			Group B		Univariate Analysis		
	N.	%	Odds Ratio (95% CI)	χ2	*p*	N.	%	Odds Ratio (95% CI)	χ2	*p*
Normal	17	37.8	0.15 (0.05–0.42)	16.57	0.0000	29	64.4	0.17 (0.039–0.63)	9.26	0.0023
Oligomenorrhea	28	62.2				16	35.6			
Normal	36	80.0				41	91.1			
Oligomenorrhea	9	20.0				4	8.9			
Short/Long	9	20.0	2.56 (0.64–12.25)	2.25	0.1338	11	24.4	1.49 (0.47–4.82)	0.60	0.4384
Normal	36	80.0				34	75.6			
Short/Long	4	8.9				8	17.8			
Normal	41	91.1				37	82.2			
Abundant/Scarce	15	33.3	2.31 (0.78–7.14)	2.86	0.0907	8	17.8	1 (0.29–3.42)	0.00	10.000
Normal	30	66.7				37	82.2			
Abundant/Scarce	8	17.8				8	17.8			
Normal	37	82.2				37	82.2			
Severe pain	13	28.9	0.66 (0.25–1.76)	0.80	0.3711	13	28.9	0.18 (0.06–0.49)	14.41	0.0001
Mild pain	32	71.1				32	71.1			
Severe pain	17	37.8				31	68.9			
Mild pain	28	62.2				14	31.1			
Severe pain	11	24.4	1.49 (0.48–4.82)	0.60	0.4384	9	20.0	0.13 (0.047–0.39)	18.22	0.0000
Mild pain	34	75.6				36	80.0			
Severe pain	8	17.8				29	64.4			
Mild pain	37	82.2				16	35.6			

**Table 3:** Inter-group comparison of ovarian function parameters in long-term survivors of lymphoma at baseline (T0) and after 12 months (T12).

Inter-GroupComparison	Group A		Group B		*p*	Group A		Group B		*p*
	T0		T0			T12		T12		
	Mean (SD)	CI 95%	Mean (SD)	CI 95%		Mean (SD)	CI 95%	Mean (SD)	CI 95%	
AMH (ng/mL)	4.23 (2.38)	3.51–4.95	4.45 (2.74)	3.62–5.28	0.3422	4.3 (2.13)	3.65–4.94	4.64 (2.64)	3.85–5.44	0.2484
FSH (mIU/mL)	10.08 (7.64)	7.77–12.38	9.94 (7.50)	7.68–12.19	0.4658	7.15 (5.43)	5.52–8.78	10.44 (6.31)	8.55–12.34	0.0047
LH (mIU/mL)	14.43 (8.22)	11.95–16.90	14.8 (8.40)	12.27–17.32	0.4169	11.20 (6.64)	9.21–13.20	15.76 (11.11)	12.42–19.10	0.0102
17-β Estradiol (pg/mL)	107.57 (45.86)	93.79–121.35	109.75 (47.45)	95.49–124.01	0.4127	100.31 (54.89)	83.81–116.80	111.83 (51.66)	96.31–127.35	0.1540
PG (ng/mL)	9.47 (4.32)	8.17–10.77	11.18 (5.55)	9.51–12.85	0.0538	11.13 (5.09)	9.60–12.66	10.92 (4.93)	9.44–12.40	0.4235
AFC of right ovary	6.22 (2.89)	5.35–7.09	7.61 (4.72)	6.18–9.03	0.0483	7.8 (2.86)	6.93–8.66	6.75 (2.73)	5.93–7.58	0.0403
AFC of left ovary	7.58 (2.64)	6.78–8.31	6.57 (2.73)	5.75–7.39	0.0406	6.93 (2.61)	6.15–7.72	7.02 (2.57)	6.25–7.79	0.4356

**Table 4:** Inter-group comparison of menstrual frequency, duration of the cycle, the amount of the menstrual cycle, dysmenorrhea, and dyspareunia in long-term survivors of lymphoma at baseline (T0) and after 12 months (T12).

		Group A		Group B		Univariate Analysis		
Variables		N.	%	N.	%	Odds Ratio (95% CI)	χ2	*p*
Frequency T0	Normal	17	37.8	29	64.4	2.98 (1.16–7.71)	6.40	0.0114
	Oligomenorrhea	28	62.2	16	35.6			
Frequency T12	Normal	36	80	41	91.1	2.56 (0.64–12.25)	2.25	0.1338
	Oligomenorrhea	9	20	4	8.9			
Duration T0	Short/Long	9	20	11	24.4	0.77 (0.25–2.35)	0.26	0.6121
	Normal	36	80	34	75.6			
Duration T12	Short/Long	4	8.9	8	17.8	0.45 (0.09–1.86)	1.54	0.2148
	Normal	41	91.1	37	82.2			
Quantity T0	Abundant/Scarce	15	33.3	8	17.8	0.43 (0.14–1.27)	2.86	0.0907
	Normal	30	66.7	37	82.2			
Quantity T12	Abundant/Scarce	8	17.8	8	17.8	1 (0.36–2.75)	0.00	10000
	Normal	37	82.2	37	82.2			
Dispareunya T0	Severe pain	13	28.9	13	28.9	1 (0.36–2.75)	0.00	10000
	Mild pain	32	71.1	32	71.1			
Dispareunya T12	Severe pain	17	37.8	31	68.9	0.27 (0.10–0.71)	8.75	0.0031
	Mild pain	28	62.2	14	31.1			
Dysmenorrea T0	Severe pain	11	24.4	9	20	1.29 (0.45–4.00)	0.26	0.6121
	Mild pain	34	75.6	36	80			
Dysmenorrea T12	Severe pain	8	17.8	29	64.4	0.11 (0.039–0.35)	20.24	0.0000
	Mild pain	37	82.2	16	35.6			

## Data Availability

All results are reported within the text.
