# Expression of Concern: Dellino et al. Effects of Oral Supplementation with Myo-Inositol and D-Chiro-Inositol on Ovarian Functions in Female Long-Term Survivors of Lymphoma: Results from a Prospective Case–Control Analysis. *J. Pers. Med.* 2022, *12*, 1536

**DOI:** 10.3390/jpm16040227

**Published:** 2026-04-20

**Authors:** Journal of Personalized Medicine Editorial Office

**Affiliations:** MDPI, Grosspeteranlage 5, 4052 Basel, Switzerland; jpm@mdpi.com

With this notice, the *Journal of Personalized Medicine* Editorial Office and Editorial Board wishes to alert readers to concerns related to this article [1]. Following publication concerns were raised regarding data integrity and reporting, including dataset structure (duplicated participant identifiers), enrollment documentation, and outcome interpretation. The investigation is being coordinated by the Editorial Office with the involvement of the Editorial Board. Once this process is complete, the Editorial Office will provide an update on this situation and announce any post-publication modification deemed to be necessary, as per MDPI’s Updating Published Papers policy.
